# Activation and Preparedness for Survivorship Care Among Survivors of Oral and Oropharyngeal Cancers

**DOI:** 10.1002/hed.28203

**Published:** 2025-06-02

**Authors:** Sharon L. Manne, Shawna V. Hudson, Janet Van Cleave, Marissa Grosso, Sara Frederick, Morgan Pesanelli, Justin Solleder, Elizabeth Handorf

**Affiliations:** ^1^ Rutgers Cancer Institute of New Jersey New Brunswick New Jersey USA; ^2^ Institute for Health, Health Care Policy and Aging Research Robert Wood Johnson Medical School New Brunswick New Jersey USA; ^3^ UTHealth Houston Cizik School of Nursing Houston Texas USA; ^4^ Rutgers School of Public Health Piscataway New Jersey USA

**Keywords:** Oral cancer, patient activation, survivorship care, survivorship preparedness

## Abstract

**Objective:**

The objectives were to characterize activation and preparedness for survivorship care and identify key sociodemographic variables, medical characteristics, care transition experiences, and psychosocial factors associated with preparedness and activation among recent oral and oropharyngeal cancer survivors.

**Methods:**

In this cross‐sectional study, 593 oral/oropharyngeal cancer survivors from cancer registries completed an online survey, and medical data were collected from the cancer registry.

**Results:**

In multivariable analyses, having Medicare or other types of non‐private insurance and having a treatment summary were associated with more activation, and lower activation was associated with being married/partnered, having a recurrence, and reporting more information needs and more depression. Older age, having a partner, Hispanic ethnicity, having Medicare insurance, and receiving a treatment summary were associated with higher preparedness for survivorship care, and being female, multiple race, employed, receiving chemotherapy, more information needs, higher fear of recurrence, and more depression were associated with less preparedness for survivorship care.

**Conclusions:**

This study identified factors associated with self‐reported activation and preparedness for survivorship care. Future research may benefit from evaluating these risk factors using a longitudinal design. If confirmed in longitudinal studies, interventions seeking to improve self‐reported activation and preparedness for survivorship care could be targeted to survivors at greater risk.

## 1 Introduction

Active engagement in managing post‐treatment care is important for successful cancer survivorship. Patient activation is broadly defined as a person's willingness to manage his or her own health as well as having an understanding of one's role in the care process and having the knowledge, skill, and confidence to manage self‐care [1, 2, 3]. In the non‐cancer context, greater activation has been associated with key health outcomes such as treatment adherence [4], higher quality of life, and better physical and psychosocial functioning [4, 5]. Among cancer survivors, research suggests that activated patients are more proactive and engaged in managing their cancer [6], believe their treatment plan reflects their values and goals [6], report lower symptom distress [7], and report higher engagement in exercise and a healthier diet [6].

A second key aspect of successful cancer survivorship is preparedness for survivorship care, typically defined as the extent to which an individual perceives she/he has sufficient information about what to expect after primary cancer treatments are complete [8]. The significance of preparedness is supported by research illustrating an association with higher perceived quality of care [9], symptom management self‐efficacy [9], and quality of life [10]. Further, studies suggest survivors who report lower levels of preparedness for survivorship care may benefit more from interventions that address symptoms and concerns than those who report higher preparedness for survivorship care [11].

The American Cancer Society estimates that there will be 58 400 new oral and oropharyngeal cancers in the US in 2024, causing 12 230 deaths [12, 13]. Survival rates have increased approximately 27% from the mid‐1970s until the 2012–2018 National Cancer Institute survey [14]. As the survivor population grows, attending to their care needs becomes increasingly important. These cancers can have a significant effect on daily functioning by impairing swallowing, taste, speaking, chewing, and comfortable movement of the head, neck, and shoulders. Common side effects include dry mouth, difficulty with mastication, speech, dental issues, loss of hearing, and/or pain [15, 16, 17]. Long‐term care needs can involve rehabilitation and pain management experts to restore and/or prevent further deterioration of function [18, 19, 20, 21]. Engagement in symptom management can improve functional outcomes [22, 23] and improve QOL and self‐efficacy [22, 23, 24, 25, 26, 27]. Recurrence rates vary depending on the type of cancer and HPV tumor type, with some studies reporting 17% to more than 80% of survivors developing a recurrence or metastatic disease within the first 3 years [28, 29, 30, 31] Based on this risk profile, the recommended surveillance regimen includes regular self‐exams [18, 19, 32], as they may lead to earlier detection of recurrence or second primary [33]. Unfortunately, adherence to post‐treatment regimen is low. Up to 80% did not engage in regular mouth care [34, 35] and over half continued to drink alcohol [36].

Due to existing constraints in oncology care provision, much of the responsibility for management of post‐treatment care falls on the survivor. For example, survivors experiencing dry mouth, difficulty swallowing, and/or weight loss make dietary changes to ensure proper nutrition. Survivors experiencing dry mouth and/or radiation to the face should perform regular mouth and dental care to reduce dry mouth and risk for dental caries. Survivors who smoke or drink alcohol should reduce use. Other self‐management tasks involve managing other medical, financial, and employment problems, and emotional distress [37, 38, 39, 40, 41].

Characterizing activation and preparedness for survivorship care and identifying the sociodemographic variables, medical characteristics, care transition experiences, and psychosocial factors associated with activation and preparedness for survivorship care can foster a greater understanding of this construct and facilitate the development of effective interventions to improve these modifiable risk factors. Unfortunately, there is very limited work evaluating activation and preparedness for survivorship care and the factors associated with them among oral and oropharyngeal cancer survivors. Mazanec and colleagues [7] found that lower activation was associated with more fatigue and depression, greater work impairment, poor perceived health, and lower quality of life among colorectal cancer survivors. Higher activation was associated with being single or living alone.

This cross‐sectional study aimed to increase our understanding of activation and preparedness for survivorship care among oral cancer survivors. The first aim was to characterize activation and preparedness for survivorship care using descriptive statistics. The second aim was to examine the association of sociodemographic variables, medical characteristics, care transition experiences, practical concerns (e.g., cancer‐related financial hardships, information needs), and psychosocial factors (e.g., depression, fear of recurrence) with activation and preparedness for survivorship care. We hypothesized that lower socioeconomic status, lack of provision of a care plan and additional follow‐up care information, fewer information needs, and lower levels of distress would be associated with lower activation and preparedness for survivorship care. The ultimate goal was to identify oral and oropharyngeal survivors at greater risk of low activation and preparedness for survivorship care to inform future self‐management interventions.

## 2 Methods

### 2.1 Eligibility

This cross‐sectional study used data from an online baseline survey from a randomized controlled trial, the Empowered Survivor trial, which evaluates a self‐management intervention for survivors of oral/oropharyngeal cancer (NCT047134). The study protocol (Pro2020000768) was reviewed and approved by the Rutgers Cancer Institute of New Jersey's IRB and IRBs at the other participating sites in accordance with the US Federal Policy for the Protection of Human Subjects. The clinicaltrials.gov registration number is NCT047113449. Inclusion criteria were (1) 18–89 years of age at initial contact; (2) diagnosed with a first primary invasive oral or oropharyngeal cancer of any stage within the past 3 years; (3) had Internet access; (4) reads English; and (5) sufficient vision to read a survey and complete an online intervention.

### 2.2 Recruitment

Participants were recruited from three state registries (New Jersey, California, Iowa) and one cancer center. New Jersey's registry confirmed eligibility, approached patients, and provided contact information to the main study site. California's registry sent contact information to the main study site. Iowa's cancer registry sent a letter to the physician informing them that the patient was eligible. If the staff did not receive a reason not to contact, they sent a letter, consent, brochure, and an envelope informing the patient of his/her eligibility and permission to release contact information to the main study site. The main study staff followed up with patients. Potential participants were sent a letter and study pamphlet. Eligibility was ascertained during a call, and eligible patients received an online consent and survey. Staff contacted participants weekly, and they were considered passive refusers if they did not return a survey after repeated calls over a one‐month period. Participants were enrolled between January 2021 and June 2024.

A total of 1809 individuals were contacted. Of these, 86 were not eligible, 145 could not be reached/incorrect contact information, 985 refused, and 593 completed the survey. Of the 292 providing a reason, the most common reason was “not interested” (70.2% 205/292). Of those eligible, acceptance was 34.4% (593/1723).

Comparisons between 593 participants and 985 refusers on available data (age, time since diagnosis, cancer location, sex, stage, minority/not, study site) indicated that oropharyngeal cancer survivors were more likely to refuse (64.5%) than oral cancer survivors (57.7%) (*t* = 6.48, *p* < 0.05). Refusers were also significantly older [*t* (1576) = 24.80, *p* < 0.001], more likely to identify as other/multiple race than white or black race (chi‐square (2) = 11.06, *p* < 0.01), carry a diagnosis of stage 3 cancer compared to any other stage [chi‐square (3) = 23.58, *p* < 0.001], and have a longer time since diagnosis [t (1576) = −24.8, *p* < 0.001]. Refusal rates were significantly higher in the Cancer Registry of Greater California (86.3%) than the New Jersey Cancer Registry (37.5%) and the Iowa Cancer Registry (21.4%) [chi‐square (3) = 427.3, *p* < 0.001]. There were no differences by sex.

### 2.3 Measures

#### 2.3.1 Sociodemographic Factors

Participants reported sex, age, education level, marital status, insurance status, race, ethnicity, income, and financial hardships in the last month. For analytic purposes, the following categories were created: Race: White, Black, Asian, multiple, other/unknown; Education: 8th grade education or less, some college/trade school, college degree or higher education; annual income was categorized as less than $30 000, $30 000–$74 999, $75 000–139 999, and $140 000 or higher; Employment status: employed or not employed; Marital status: partnered or not partnered, insurance type: private, Medicare, Medicaid, other, or unknown. Participants rated current financial strain using one item: “During the past 4 weeks, did you have adequate financial resources to meet the daily needs of you and your family?” (yes/no). The state‐level Area Deprivation Index (ADI) was calculated. ADI is based on a measure originally created by the Heath RSA that was adapted and validated to the Census Block Group neighborhood level [42, 43, 44, 45]. The ADI ranks neighborhoods by socioeconomic disadvantage [44, 45], with a ranking of 1 to 100 as a metric of neighborhood socioeconomic disadvantage, with 100 being the most disadvantaged neighborhoods nationwide. Twenty‐five points were added for Medicare and Medicaid dual eligibility, which is individual‐level measure of disadvantage [44]. To calculate a state ADI, census data block group data were ranked in percentiles from 1 to 10 (1 = least disadvantaged neighborhood within the residential state‐10 = most disadvantaged neighborhood).

#### 2.3.2 Medical/Clinical Factors

Medical variables included time since diagnosis, recurrence status, and treatments received collected from the patient's medical record, as well as self‐reported comorbidities [46]. Tumor location and cancer stage were extracted from registry data. Cancer stage was categorized as 0/1, 2, 3, and 4. Tumor types were characterized as oral or oropharyngeal cancer. Fourteen subscales of the European Organization for Research and Treatment of Cancer‐Quality of Life Questionnaire‐Head and Neck‐43 [47] were used to assess head and neck cancer post‐treatment physical symptoms and side effects. A mean standard score was used (range = 1–100), *α* = 0.88.

#### 2.3.3 Care Transition Practices

Participants were asked if they received a written summary from their doctor(s) that mentioned details of the treatment they received and provided other important details regarding their cancer care at the end of treatment (*yes*/*no*).

#### 2.3.4 Psychosocial Factors

##### 2.3.4.1 Information Needs

This 23‐item scale assessed the desire for more information about oral cancer‐specific topics (e.g., managing dry mouth). Response choices were *yes/no* and participants were asked to consider needs “at this time.” This scale was adapted from the Health‐Related Topics section of the FOCUS [48] used in our prior work [49]. A total number of needs was calculated, and a higher score indicates more needs. *α* = 0.95.

##### 2.3.4.2 Concerns About Recurrence [51]

The global fear scale of the Concerns about Recurrence Scale (CARS) [50] assessed current worry about a recurrence of oral cancer (1 = *It does not upset me at all*, 7 = *It makes me extremely upset*). This scale has demonstrated strong internal consistency [51, 52] as well as convergent validity [50]. A mean score was calculated. α = 0.93 [51].

##### 2.3.4.3 Depressive Symptoms

The Patient Health Questionnaire (PHQ‐9) [53] assessed self‐reported diagnostic criteria for depression. It is a widely used measure and has strong psychometrics [54]. Items are scored on a Likert scale (1 = *not at all*, 2 = *several days*, 3 = *more than half the days*, 4 = *nearly every* day), and participants were instructed to rate how they felt in the last 2 weeks. Scores from 0 to 4 indicate no/mild depression, 5 to 9 indicate mild, 10 to 14 indicate moderate, 15 to 19 indicate moderately severe, and 20 to 27 indicate severe depression. *α* = 0.89.

### 2.4 Outcomes

#### 2.4.1 Activation

Activation was defined as understanding one's role in the care process and having the knowledge, skill, and confidence to manage cancer self‐care. We adapted the Patient Activation Measure [1, 55] for this study to focus on the management of oral and oropharyngeal cancer by removing one question, “I know the nature and causes of my health condition.” as this question did not seem appropriate for a cancer diagnosis. The scale contained 12 items (sample item: “When all is said and done, I am the person who is responsible for managing my head and neck cancer care”). The response scale was modified to a four‐point Likert (1 *= Disagree Strongly*, 2 *= Disagree*, 3 *= Agree*, 4 *= Agree Strongly*). A mean score was calculated. This scale has demonstrated satisfactory construct validity *α* = 0.90 [55]. Items are shown in Table 1.

**TABLE 1:** Activation and preparedness for survivorship care measures.

Activation scale items
When all is said and done, I am the person who is responsible for managing my head and neck cancer care.
2 Taking an active role in my head and neck cancer care is the most important factor in determining my health and ability to function.
3 I am confident that I can take actions that will help prevent or minimize some symptoms or problems associated with my head and neck cancer care.
4 I know what each of my prescribed medications are, if there are any for my head and neck cancer.
5 I am confident that I can tell when I need to go get medical care for my cancer and when I can handle health problems myself.
6 I am confident I can tell my health care provider concerns I have for my cancer even when he or she does not ask.
7 I am confident that I can follow through on my head and neck cancer home care (swallowing or muscle strength exercises, managing dry mouth, nutrition, oral self‐examinations).
8 I know the recommended self‐care practices for my head and neck cancer.
9 I have been able to maintain the lifestyle changes for my health that I have made.
10 I know how to prevent further problems with my head and neck cancer.
11 I am confident I can figure out solutions when new situations or problems arise with my head and neck cancer.
12 I am confident that I can maintain lifestyle changes like diet and exercise even during times of stress.

Preparedness for survivorship scale items
I have been given a sufficient amount of information about head and neck cancer survivorship
2 The information I received to date about head and neck cancer survivorship has been easy to understand
3 The information I received to date about head and neck cancer survivorship has been helpful to me.
4 The information I received to date about head and neck cancer survivorship has covered the issues and needs I have
5 The information I received to date about head and neck cancer survivorship has addressed how to manage my symptoms
6 The information I received to date about head and neck cancer survivorship has covered how to look for signs of oral cancer.
7 The information I received to date about head and neck cancer survivorship has covered which exercises are helpful for me to perform on a daily basis to increase or maintain functioning.
8 The information I received to date about head and neck cancer survivorship has outlined a follow‐up care appointment schedule.
9 How satisfied are you with *the amount of information* you received to date about head and neck cancer survivorship
10 How satisfied are you with the way the information about head and neck cancer survivorship was presented to you?

#### 2.4.2 Preparedness for Survivorship Care

This construct was defined as the extent to which individuals perceived they have sufficient information about what to expect after cancer treatments are completed. Ten items adapted from Manne and colleagues [56, 57] assessed whether information received about survivorship care was sufficient, easy to understand, and helpful; whether it addressed needs, symptom management, and looking for signs of cancer; and whether it outlined follow‐up care. Items were rated on a Likert scale (Questions 1–8: 1 *= Strongly Disagree*, 2 *= Moderately Disagree*, 3 *= Moderately Agree*, 4 *= Strongly Agree*. Questions 9–10: 1 *= Not at all Satisfied*, 2 = *A little Satisfied*, 3 = *Moderately Satisfied*, 4 = *Very Satisfied*, 5 = *Extremely Satisfied*). A mean score was calculated. α = 0.95. Items are shown in Table 1.

### 2.5 Approach to Analyses

Participants with missing data for activation or preparedness for survivorship care were excluded from the respective analysis. Covariate missingness was tabulated and described. Where possible, we used a missing category approach for covariates. We conducted multivariable linear regression analyses to determine what demographic, disease, and psychological factors were associated with activation and preparedness for survivorship care. We used robust standard errors via Generalized Estimating Equations to account for within‐site correlation. Due to the interrelationship between education, income, and financial resources, only one of these variables could be included in the multivariable model. We selected income, based on the Quasi Information Criterion for model fit. All other variables of interest were simultaneously included in the model. The proportion of variability in the outcome accounted for by the model was measured using marginal R‐squared. A *p*‐value of < 0.05 was considered statistically significant. Standardized effects were calculated by standardizing both the outcomes and continuous predictors. R Software (version 4.1) was used for regression analyses.

## 3 Results

### 3.1 Descriptive Statistics

Descriptive data are shown in Table 2. Participants were primarily male (71.7%) and white non‐Hispanic (83.8%). Approximately half had a college level or higher education (51.3%) and about half (51.6%) were currently employed. About a third (31.3%) lived in the most deprived areas of the state in which they resided. About 11% reported an annual income lower than $30 000. About half (54.5%) had private insurance. More than half were diagnosed with oropharyngeal cancer (61.4%) and with early‐stage cancer (55.1%). Average time since diagnosis was slightly less than 2 years (range = 0.53–3.37). Relatively few (5.6%) had a recurrence during this interval. About 72% reported receiving a written summary of treatment and details about recommended post‐treatment cancer care.

**TABLE 2:** Demographic and medical characteristics of the sample (*N* = 593).

Variable	*N* (%)	M (SD)
Age (years)		62.14 (10.63)
Sex		
Male	425 (71.7%)	
Female	168 (28.3%)	
Race		
White	505 (85.2%)	
Black	33 (5.6%)	
Asian	21 (3.5%)	
Multiple	20 (3.4%)	
Other/unknown	14 (2.4%)	
Ethnicity		
Non‐Hispanic	556 (93.8)	
Hispanic	32 (5.4)	
Unknown	5 (0.8)	
Education		
Up to 12th grade	112 (18.9%)	
Some college/trade school	175 (29.5%)	
College degree or higher	304 (51.3%)	
Uknown	2 (0.3%)	
Income		
< $30,000	67 (11.3%)	
$30 000–$74 999	153 (25.8%)	
$75 000–$139 999	171 (28.8%)	
$140 000 or more	195 (32.9%)	
Unknown	7 (1.2%)	
Marital status		
Partnered	437 (73.7%)	
Not Partnered	156 (26.3%)	
Employment		
Employed	306 (51.6%)	
Not employed	287 (48.4%)	
Insurance Type		
Private	323 (54.5%)	
Medicare	202 (34.1%)	
Medicaid	33 (5.6%)	
Other	26 (4.4%)	
Unknown	9 (1.5%)	
Area deprivation index (state)		
1 (least deprived)	78 (13.2%)	
2	70 (11.8%)	
3	57 (9.6%)	
4	60 (10.1%)	
5	64 (10.8%)	
6	72 (12.2%)	
7	43 (7.3%)	
8	62 (10.5%)	
9	48 (8.1%)	
10 (most deprived)	38 (6.4%)	
Adequate finances to meet daily needs		
Yes	536 (90.7%)	
No	55 (9.3%)	
Cancer location		
Oral cavity	229 (38.6%)	
Oropharyngeal	364 (61.4%)	
Cancer stage		
0/1	327 (55.1%)	
2	125 (21.1%)	
3	47 (7.9%)	
4	94 (15.9%)	
Recurrence (yes)	33 (5.6%)	
Time since diagnosis (years)		1.96 (0.73)
Treatments		
Surgery (yes)	342 (57.7%)	
Radiation (yes)	412 (69.5%)	
Chemotherapy (yes)	266 (44.9%)	
Comorbidities		0.73 (1.00)
Received treatment summary		
Yes	425 (71.7%)	
No	162 (27.3%)	
Unknown	6 (1.0%)	

Table 2 (bottom row) presents the overall descriptive statistics for activation and preparedness for survivorship care. The average activation score (*M* = 3.26) corresponded to “agree” on the 4‐point scale, and the lowest level of activation ratings were obtained on the item “I know how to prevent further problems with my head and neck cancer” (*M* = 2.77). Average preparedness (*M* = 3.1) corresponded to “moderately agree” (3 = *moderately* on a 4‐point scale). The lowest preparedness for survivorship care ratings were obtained on the item “has covered how to look for signs of oral cancer,” (*M =* 2.62, SD = 1.01, 3 = *moderately agree*). Correlations between variables included in the analyses are shown in Table 3. The correlation between activation and preparedness was significant (*r* = 0.43, *p* < 0.001).

**TABLE 3:** Correlations and descriptive information on outcome and predictor variables.

	1.	2.	3.	4.	5.	6.
Activation	1.00					
2 Preparedness for survivorship care	0.43**	1.00				
3 Cancer symptoms	−0.27**	−0.23**	1.00			
4 Information needs	−0.29**	−0.33**	0.50**	1.00		
5 Fear of recurrence	−0.21**	−0.28**	0.37**	0.31**	1.00	
6 Depressive symptoms	−0.33**	−0.31**	0.58**	0.36**	0.44**	1.00
*M*	3.26	3.11	18.97	8.95	2.91	4.22
SD	0.45	0.83	15.67	6.73	1.44	5.10

** *p* < 0.001.

Table 2 also presents scores on measures of the symptoms and psychosocial correlates. In terms of physical symptoms, the average was 18.97 (SD = 15.67). The highest rated symptom was dry mouth (*M* = 43.8, SD = 31.69). The most‐commonly reported information needs were: how to do an oral self‐exam (66.1%), what a suspicious spot or lesion looks like (64.2%), symptoms that should prompt calling the doctor (61.2%), and what late and long‐term side effects of oral cancer treatment to expect (59.5%). Over half of participants (63.5%) were in “no to mild” range on the depression measure, 22.8% were in “mild” depression range, 8.3% were in “moderate” range, 3.2% were in “moderately severe” range, and 2.2% were in “severe” range. Fear of recurrence was relatively low (item *M* = 2.91, SD = 1.44, scale 0–7). Comparisons with studies of other cancer survivor populations using the same measure indicate the current sample reported higher general fear than survivors of mixed types of cancer (*M* = 1.30, SD = 1.0) [58].

### 3.2 Results of Multivariable Analyses Predicting Activation

The multivariable model for activation is shown in Table 4. Having Medicare or other insurance was significantly associated with higher activation than having private insurance (β = 0.052, 95% CI 0.015–0.089, β = 0.130, 95% CI 0.017–0.244, respectively). Being married or partnered was associated with lower activation (β = −0.013, 95% CI −0.018 to −0.007). A recurrence was associated with lower activation (*β* = −0.088, 95% CI −0.156 to −0.020). Receiving a treatment summary was associated with significantly higher activation (β = 0.170, 95% CI 0.129–0.210). More information needs and higher levels of depression were associated with lower activation (β = −0.014, 95% CI −0.017 to −0.010, β = −0.022, 95% CI −0.026, −0.018, respectively). The marginal R^2^ statistic was 0.23, so the model accounted for 23% of the variance in activation.

**TABLE 4:** Multivariable regression model predicting activation.

Variable	Estimate	Std err	Std estimate	95% CI (Est)		*p*
(Intercept)	**3.297**	**0.035**		**3.229**	**3.365**	**< 0.001**
Age (per 10 year increase)	0.000	0.013	−0.001	−0.025	0.024	0.976
Female gender	0.007	0.028	0.015	−0.047	0.061	0.804
Black (vs White)	0.112	0.065	0.249	−0.015	0.239	0.084
Asian (vs White)	−0.065	0.079	−0.143	−0.219	0.090	0.413
Multiple (vs White)	−0.020	0.034	−0.043	−0.086	0.047	0.566
Other/unknown (vs White)	−0.026	0.040	−0.057	−0.103	0.052	0.518
Hispanic (vs non‐Hispanic)	0.221	0.113	0.49	−0.001	0.443	0.051
Income 30–74.9 k (vs < 30 k)	−0.035	0.071	−0.078	−0.175	0.104	0.621
Income 75–139.9 k (vs < 30 k)	0.090	0.081	0.201	−0.068	0.249	0.263
Income 140 k + (vs < 30 k)	0.089	0.048	0.197	−0.005	0.183	0.064
Married or partnered (vs single)	**−0.013**	**0.003**	**−0.028**	**−0.018**	**−0.007**	**< 0.001**
Employed full or part time	0.004	0.009	0.008	−0.015	0.022	0.690
Medicare insurance (vs private)	**0.052**	**0.019**	**0.116**	**0.015**	**0.089**	**0.006**
Medicaid insurance (vs private)	0.146	0.087	0.324	−0.025	0.317	0.093
Other insurance (vs private)	**0.130**	**0.058**	**0.289**	**0.017**	**0.244**	**0.025**
ADI (State)	0.009	0.007	0.053	−0.004	0.022	0.197
Oropharyngeal (vs oral cavity)	−0.007	0.023	−0.015	−0.053	0.039	0.770
Stage II (vs 0/I)	−0.028	0.065	−0.063	−0.156	0.099	0.663
Stage III (vs 0/I)	−0.050	0.074	−0.111	−0.195	0.095	0.499
Stage IV (vs 0/I)	−0.026	0.032	−0.057	−0.088	0.037	0.422
Recurrence	**−0.088**	**0.035**	**−0.195**	**−0.156**	**−0.020**	**0.012**
Time since diagnosis (years)	0.001	0.011	0.002	−0.020	0.023	0.902
Had surgery	0.013	0.012	0.028	−0.011	0.037	0.292
Had radiation	−0.008	0.017	−0.019	−0.042	0.025	0.626
Had chemotherapy	−0.042	0.028	−0.093	−0.097	0.013	0.134
Comorbidities	−0.005	0.015	−0.011	−0.035	0.025	0.753
Received treatment summary	**0.170**	**0.021**	**0.377**	**0.129**	**0.210**	**< 0.001**
Symptoms	0.001	0.001	0.028	−0.001	0.003	0.474
Information needs	**−0.014**	**0.002**	**−0.206**	**−0.017**	**−0.010**	**< 0.001**
Fear of recurrence	−0.012	0.015	−0.04	−0.042	0.017	0.413
Depression	**−0.022**	**0.002**	**−0.246**	**−0.026**	**−0.018**	**< 0.001**

*Note:* The missing category for race, income, insurance, receipt of treatment summary, and depression score is included in the model but not shown. Bolded values indicated that the *p* value for that variable was statstically signficant.

### 3.3 Results of Multivariable Analyses Predicting Preparedness for Survivorship Care

The multivariable model for preparedness for survivorship care is shown in Table 5. Results indicated that greater preparedness for survivorship care was significantly associated with older age (β = 0.065 per each additional 10 years of age, 95% CI 0.042–0.088) having a partner (β = 0.057, 95% CI 0.041–0.074), having Hispanic ethnicity or reporting “other” race compared with White race (β = 0.347, 95% CI 0.291–0.402, β =0.128, 95% CI 0.009–0.247, respectively) having Medicare insurance (versus private insurance, β = 0.041, 95% CI 0.015–0.067), and receipt of a treatment summary (β = 0.585, 95% CI 0.524–0.646). Lower preparedness for survivorship care was significantly associated with being female (β = −0.095, 95% CI −0.125 to −0.066), reporting multiple races (β = −0.184, 95% CI −0.304 to −0.064), being currently employed (β = −0.056, 95% CI −0.111 to −0.002), having recurrent disease (β = −0.106, 95% CI −0.195 to −0.018), receiving chemotherapy (β = −0.098, 95% CI −0.195 to −0.002), more information needs (β = −0.026, 95% CI −0.029 to −0.023), higher fear of recurrence (β = −0.065, 95% CI −0.105 to −0.024) and more depressive symptoms (β = −0.023, 95% CI −0.026 to −0.020). The marginal R^2^ statistic was 0.29, so the model accounted for 29% of the variance in preparedness for survivorship care.

**TABLE 5:** Multivariable regression model predicting preparedness for survivorship care.

Variable	Estimate	Std err	Std estimate	95% CI (Est)		*p*
(Intercept)	**2.767**	**0.105**		**2.561**	**2.973**	**< 0.001**
Age (per 10 year increase)	**0.065**	**0.012**	**0.065**	**0.042**	**0.088**	**< 0.001**
Female gender	**−0.095**	**0.015**	**−0.081**	**−0.125**	**−0.066**	**< 0.001**
Black (vs White)	−0.018	0.085	0.058	−0.185	0.149	0.833
Asian (vs White)	−0.146	0.125	−0.228	−0.390	0.099	0.243
Multiple (vs White)	**−0.184**	**0.061**	**−0.228**	**−0.304**	**−0.064**	**0.003**
Other/unknown (vs White)	**0.128**	**0.061**	**0.418**	**0.009**	**0.247**	**0.035**
Hispanic (vs non‐Hispanic)	**0.347**	**0.028**	**0.065**	**0.291**	**0.402**	**< 0.001**
Income $30–74.9 k (vs < 30 k)	0.085	0.054	0.096	−0.020	0.190	0.111
Income $75–139.9 k (vs < 30 k)	0.085	0.057	0.121	−0.027	0.196	0.136
Income $140 k + (vs < 30 k)	−0.010	0.061	−0.015	−0.129	0.108	0.867
Married or partnered (vs single)	**0.057**	**0.009**	**0.106**	**0.041**	**0.074**	**< 0.001**
Employed full or part time	**−0.056**	**0.028**	**−0.059**	**−0.111**	**−0.002**	**0.043**
Medicare insurance (vs private)	**0.041**	**0.013**	**0.090**	**0.015**	**0.067**	**0.002**
Medicaid insurance (vs private)	0.242	0.152	0.348	−0.055	0.540	0.110
Other insurance (vs private)	−0.039	0.052	−0.021	−0.140	0.062	0.454
ADI (State)	−0.001	0.007	0.009	−0.016	0.013	0.884
Oropharyngeal (vs oral cavity)	0.104	0.093	0.144	−0.077	0.286	0.260
Stage II (vs 0/I)	−0.036	0.025	−0.042	−0.086	0.013	0.150
Stage III (vs 0/I)	0.063	0.077	0.094	−0.087	0.213	0.411
Stage IV (vs 0/I)	−0.027	0.057	−0.027	−0.139	0.086	0.644
Recurrence	**−0.106**	**0.045**	**−0.123**	**−0.195**	**−0.018**	**0.019**
Time since diagnosis (years)	−0.028	0.030	−0.028	−0.088	0.031	0.353
Had surgery	−0.016	0.034	−0.044	−0.083	0.051	0.643
Had radiation	0.038	0.085	0.048	−0.129	0.205	0.656
Had chemotherapy	**−0.098**	**0.049**	**−0.139**	**−0.195**	**−0.002**	**0.046**
Comorbidities	−0.006	0.010	0.012	−0.025	0.013	0.542
Received treatment summary	**0.585**	**0.031**	**0.701**	**0.524**	**0.646**	**< 0.001**
Symptoms	0.001	0.001	0.026	−0.001	0.002	0.293
Information needs	**−0.026**	**0.002**	**−0.216**	**−0.029**	**−0.023**	**< 0.001**
Fear of recurrence	**−0.065**	**0.021**	**−0.122**	**−0.105**	**−0.024**	**0.002**
Depression	**−0.023**	**0.002**	**−0.141**	**−0.026**	**−0.020**	**< 0.001**

*Note:* The missing category for race, income, insurance, receipt of treatment summary, and depression score is included in the model but not shown. Bolded values indicated that the *p* value for that variable was statstically signficant.

## 4 Discussion

Survivors of oral and oropharyngeal cancers typically manage myriad medical and psychosocial effects from the disease and its treatment. Understanding the recommended self‐management tasks, having accurate information about expected symptoms and recommended follow‐up care, and possessing sufficient skills and confidence to manage care may enhance care quality and outcomes. This study described activation and preparedness for survivorship care and identified some key demographic, medical, care transition practices, and psychosocial factors associated with activation and preparedness for survivorship care in a sample of oral and oropharyngeal survivors who had completed treatment in the last 3 years.

Survivors reported moderate activation levels. Most felt responsible for managing care, took an active role, felt able to express concerns, minimize symptoms, and knew what medications to take. They felt less knowledgeable about how to prevent further problems, recommended home care, and managing new problems. Levels of activation were consistent with prior work focusing on breast and prostate survivors [59] and studies examining a range of cancers [60] but higher than a prior study of lymphoma survivors [61]. Overall, survivors felt moderately prepared, but least prepared about identifying a recurrence. Findings are similar to studies using the same measure in other cancer survivor populations [57, 61] as well as prior work using a different measure in a mixed sample of cancer patients [9]. Taken together, our findings suggest specific gaps in knowledge and skills, specifically regarding recommended cancer‐related home care, how to identify a recurrence, and how to handle new symptoms.

### 4.1 Activation

Some of the activation model findings are consistent with prior work with other survivor populations and the general literature, whereas other findings are not. Prior studies [7, 59, 61] also reported that single/unmarried survivors were more activated. A potential explanation is that married survivors have the assistance of a spouse, whereas those without a partner carry more responsibility for managing their care. Prior work has also suggested that providers' care transition practices are associated with more activation [59, 61]. Greater information needs [61] have been associated with lower activation in other survivor populations. The limited prior work regarding the association between depressive symptoms is mixed, with some studies reporting an association [7] and others not [61]. Our findings suggest that depressive symptoms are related to diminished activation and preparation. Depression has been associated with lower activation among individuals in the general population [62], individuals with other serious illnesses such as multiple sclerosis [63] and HIV [64], and primary care patients [65]. Although we cannot infer causation, it is possible that interventions targeting either activation or depression may impact both outcomes. Although not previously reported, recurrence may create more concerns about how to recognize and manage survivorship care. Finally, it was surprising that survivors carrying Medicare/other insurance reported higher activation than survivors with private insurance. It is difficult to propose an explanation for this finding, and future studies can further examine this association. Generally, effect sizes of predictors for activation were modest, with the largest standardized effect size being 0.377 for receipt of treatment summary, with other effect sizes smaller than 0.25.

### 4.2 Preparedness for Survivorship Care

Some findings from our preparedness for survivorship care model were consistent with prior work. More preparedness for survivorship care has been reported among older survivors [57] employed survivors [61] and survivors receiving a treatment summary [9, 61]. Lower preparedness for survivorship care has been documented among survivors with more information needs [57, 61] and more depressive symptoms [9]. It is possible that younger survivors are more likely to work and therefore have less time for preparation and therefore more information needs. Surprisingly, our findings indicated higher preparedness for Hispanics and lower preparedness for survivors identifying as multiple races. Limited prior work comparing Hispanics and non‐Hispanics has not demonstrated differences in preparedness for survivorship care [66]. However, because the percentages of participants identifying as Hispanic and multiple races were small, future research with greater racial and ethnic diversity should explore this relationship. Prior studies have not evaluated insurance status as a correlate of preparedness for survivorship care [9, 57, 66, 67, 68, 69]. It is possible that this association was due to the older age of survivors enrolled in Medicare as compared with survivors who have private insurance. Like activation, effect sizes for covariates were generally modest, with the exception being receipt of a treatment summary, with a large standardized effect size of 0.701. Other significant effect sizes were moderate to small. The pattern of correlates of preparedness across the literature is uneven.

It is interesting to note that we did not find an association between income and area deprivation and activation or preparedness for survivorship care. Due to the interrelationship between education, income, and financial resources, only income was included in the models, and education and financial hardship were not included. In prior work among survivors of other cancers, financial hardship has been associated with less preparedness for survivorship care [57] and greater area deprivation has been associated with less activation [61]. As higher neighborhood deprivation has been associated with lower quality of life and head and neck disability [70], future work should continue to examine deprivation and survivorship outcomes.

### 4.3 Strengths, Limitations, and Conclusions

Strengths are the large sample, relatively equal representation of oral and oropharyngeal cancer survivors, and recruitment from cancer registries from East Coast, Midwest, and West Coast. One limitation is the cross‐sectional methodology, which precludes the ability to draw causal or temporal inferences. The sample consisted primarily of non‐Hispanic white, male, married, higher income, and insured survivors diagnosed with early‐stage oral cancers. Finally, non‐respondents were older, had a longer time since diagnosis, were more likely to come from California, and more likely to have oropharyngeal cancer relative to participants. These differences between participants and nonparticipants may have biased the findings. Future studies may benefit from enhanced recruitment strategies targeting these groups. Also, there is limited information about the validity of the preparedness for survivorship care measure. Finally, effect sizes for the predictors were modest, with only the receipt of a treatment summary having a large effect size on preparedness.

In conclusion, this study identified risk factors and characteristics associated with activation and preparedness for survivorship care that may be useful for clinicians working with the oral cancer survivor population. Future research may benefit from evaluating these risk factors using a longitudinal design. If confirmed in longitudinal studies, interventions seeking to improve activation and preparedness for survivorship care could be targeted to survivors at greater risk.

## Author Contributions

Sharon Manne, Shawna Hudson, and Janet Van Cleave contributed to the study conception and design. Material preparation and data collection were performed by Morgan Pesanelli, Sara Frederick, and Marissa Grosso. Data analysis was performed by Beth Handorf and Sharon Manne. Manuscript preparation was done by Sharon Manne, Justin Solleder, and Beth Handorf. The first draft of the manuscript was written by Sharon Manne, and all authors commented on previous versions of the manuscript.

## Ethics Statement

The study protocol (Pro2020000768) was reviewed and approved by the Rutgers Cancer Institute's IRB and IRBs at the other participating sites in accordance with the US Federal Policy for the Protection of Human Subjects.

## Consent

Informed consent was obtained for all participants, and this information is in the paper.

## Conflicts of Interest

The authors declare no conflicts of interest.

## Data Availability

The data that support the findings of this study are available on request from the corresponding author. The data are not publicly available due to privacy or ethical restrictions.
